# LincRNA-p21 activates endoplasmic reticulum stress and inhibits hepatocellular carcinoma

**DOI:** 10.18632/oncotarget.4661

**Published:** 2015-07-20

**Authors:** Yang Ning, Fu Yong, Zhang Haibin, Sima Hui, Zhu Nan, Yang Guangshun

**Affiliations:** ^1^ Hepatobiliary Surgery Department V, Eastern Hepatobiliary Surgery Hospital, Second Military Medical University, Shanghai, China

**Keywords:** lincRNA-p21, hepatocellular carcinoma, ER stress, sorafenib

## Abstract

LincRNA-p21 is a downstream long non-coding RNA (lncRNA) transcript of p53. LincRNA-p21 serves as a repressor in p53-dependent transcriptional responses and participates in diverse biological processes, including apoptosis, cell cycle, metabolism and pluripotency. However, the role of lincRNA-p21 in human hepatocellular carcinoma remains to be defined. Here in this work, we demonstrated that lincRNA-p21 acted as a tumor suppressive lncRNA in human hepatocellular carcinoma. We firstly found the downregulation of lincRNA-p21 level in human hepatocellular carcinoma tissues, and showed that low expression of lincRNA-p21 was associated with high disease stage and predicted poor survival. Further we showed that lincRNA-p21 knockdown promoted proliferation and colony formation of HepG2, Huh7 and Bel-7042 cells *in vitro*, while lincRNA-p21 overexpression obtained oppose results. Using tumor xenograft experiments, we also demonstrated that lincRNA-p21 inhibited HepG2 cell growth *in vivo* and lincRNA-p21 contributed to sorafenib-induced growth regression of HepG2 cell *in vivo*. Further mechanism analysis revealed that lincRNA-p21 promoted ER stress both in *vitro* and *in vivo*, which facilitated apoptosis of hepatocellular carcinoma cells. Finally, we demonstrated that ER stress accounted for lincRNA-p21 effects on apoptosis, proliferation and *in vivo* growth of hepatocellular carcinoma. These findings implicate that lincRNA-p21 is a potential prognostic factor and therapeutic target for human hepatocellular carcinoma.

## INTRODUCTION

Hepatocellular carcinoma (HCC) causes more than 500,000 deaths each year worldwide [1]. However, measures aiming at preventing HCC development in these patients are limited [2]. In addition, current therapies for HCC often obtain poor long-term outcome due to drug resistance with elusive mechanisms [3]. These facts prompt us to identify novel molecular mechanisms for hepatocellular carcinoma development and drug resistance.

Endoplasmic reticulum (ER) stress has emerged as a major site of cellular homeostasis regulation, particularly in the unfolded protein response, which plays a major role in cancer and many other diseases [4]. Several drugs that activate ER stress have been approved for preclinical and clinical use. These drugs include sorafenib, eeyarestatin, 17-AAG, radicicol, and MAL3-101 [5]. The contribution of ER stress to HCC has been proposed repeatedly [6, 7], however, high activation of ER stress by sorafenib could induce apoptosis and growth arrest in established or advanced HCC [8, 9]. Similar situation was observed for marine prostanoid, curcumin, and genistein [10–12]. The mechanism by which sorafenib induces ER stress and HCC cell apoptosis is still elusive.

Long non-coding RNAs (lncRNAs) have recently been found to be pervasively transcribed in the human genome. Aberrant expression of several lncRNAs was found to be involved in the carcinogenesis of human HCC [13, 14]. The p53-regulated long noncoding RNA lincRNA-p21 has been proposed to act in trans *via* several mechanisms ranging from repressing genes in the p53 transcriptional network to regulating mRNA translation and protein stability [15]. The physiological and pathological functions of lincRNA-p21 were identified gradually. For instance, Yang *et al*. [16] reported lincRNA-p21 as a regulator for the Warburg effect and also implicated lincRNA-p21 as a valuable therapeutic target for cancer. Wu *et al*. [17] showed that lincRNA-p21 regulated neointima formation, vascular smooth muscle cell proliferation, apoptosis, and atherosclerosis by enhancing p53 activity. LincRNA-p21 function in pluripotency was also been identified [18, 19]. Here in the present work, we identified lincRNA-p21 as a negative regulator for HCC development and drug resistance.

## RESULTS

### LincRNA-p21 is down-regulated in human hepatocellular carcinoma

**Figure 1:**
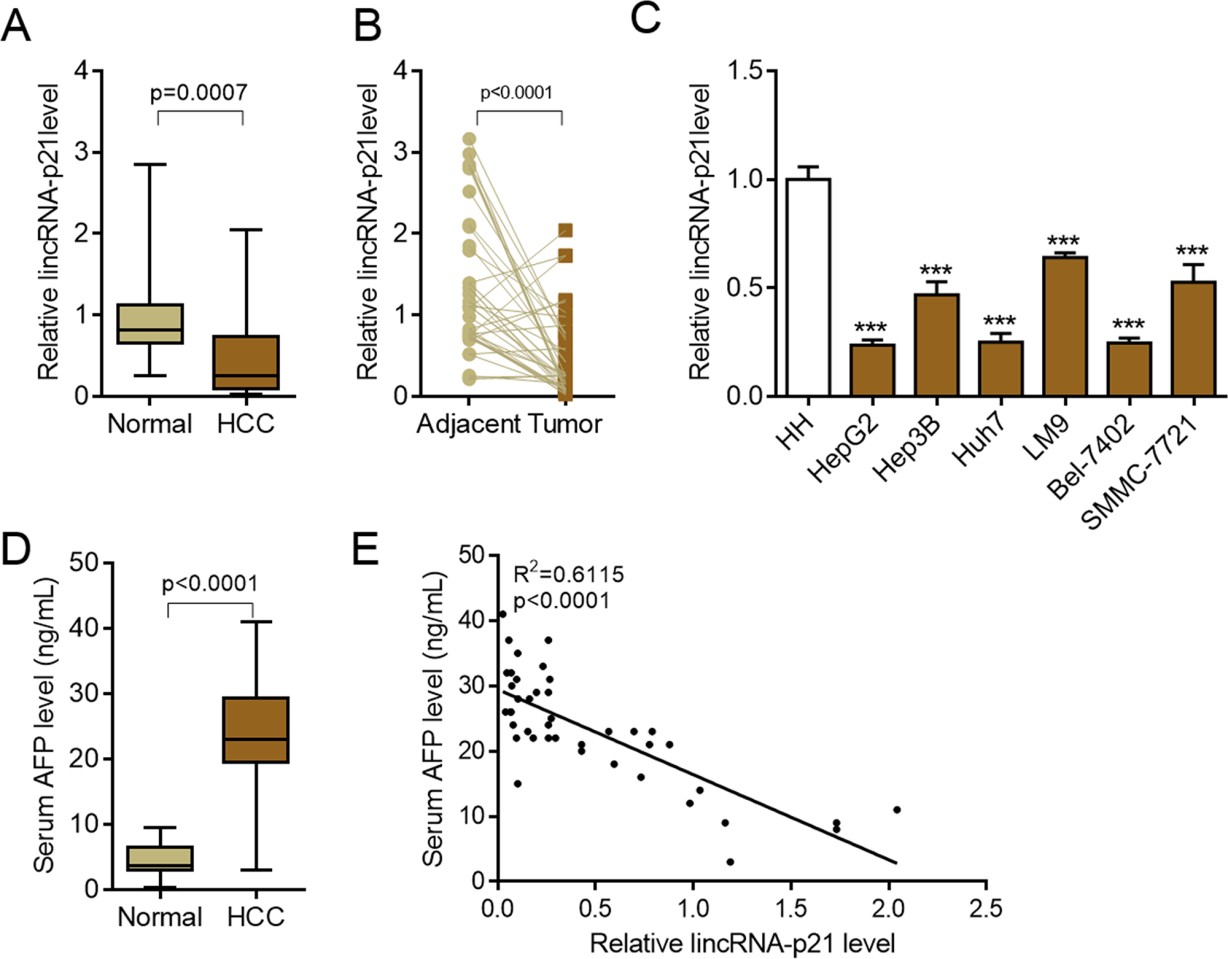
LincRNA-p21 is down-regulated in hepatocellular carcinoma **A.** LincRNA-p21 expression in human hepatocellular carcinoma (HCC, *n* = 42) or control normal liver tissues (*n* = 22). **B.** LincRNA-p21 levels in adjacent normal liver tissues and tumor tissues in HCC. *n* = 40. Paired Student's *t* test was performed to analyze the data. **C.** LincRNA-p21 expression in normal human hepatocytes (HH) and liver cancer cell lines (HepG2, Hep3B, Huh7, LM9, Bel-7042, SMMC-7721). ***p < 0.001 *vs*. HH **D.** Serum levels of alpha-fetoprotein (AFP) in HCC patients (*n* = 42) or normal tissue donors (*n* = 24). **E.** Correlation between serum AFP and lincRNA-p21 level (*n* = 42).

**Figure 2:**
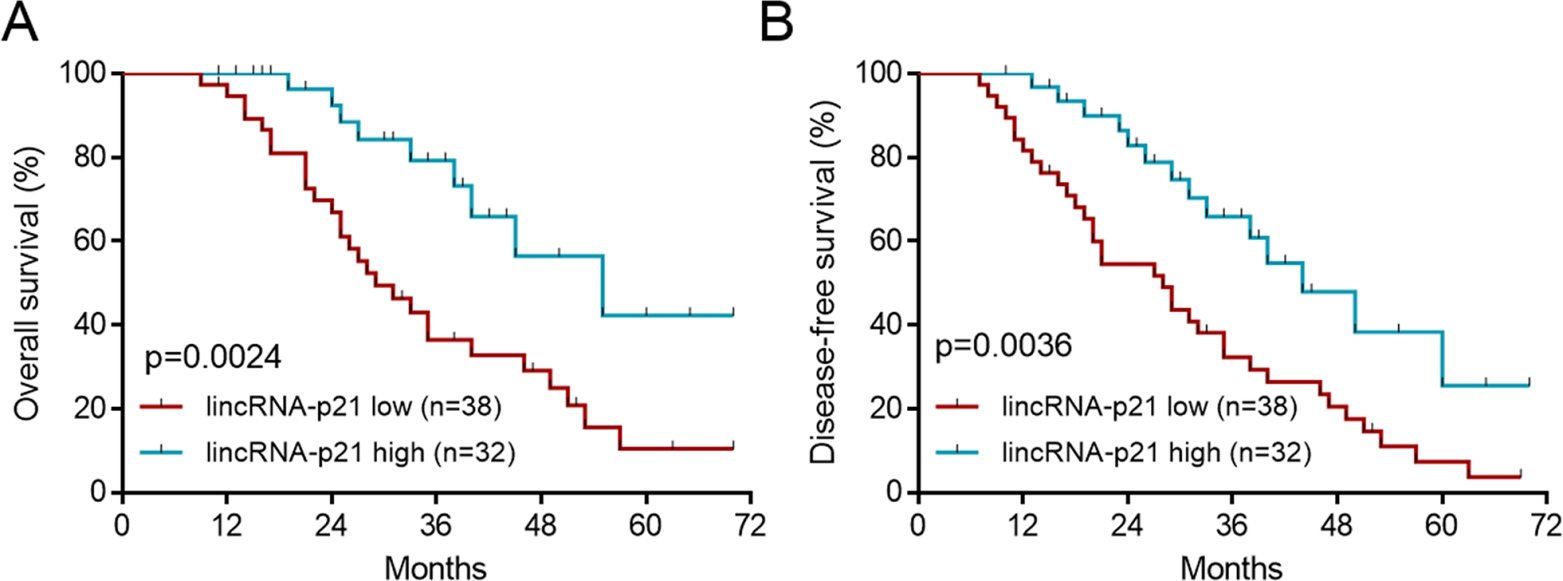
LincRNA-p21 low expression predicts poor survival The patients were divided to lincRNA-p21 high group and lincRNA-p21 low group by the mean value of total 70 HCC patients. **A.** Low lincRNA-p21 level predicts poor overall survival in HCC patients. **B.** Low lincRNA-p21 predicts poor disease-free survival in HCC patients.

To investigate the potential functions of lincRNA-p21 in human hepatocellular carcinoma (HCC), we firstly tested the expression profile of this lincRNA in normal liver tissues and tissues from HCC. The results showed that lincRNA-p21 level was significantly reduced in tissues from HCC than that from normal liver tissues (Figure 1A). We also tested the expression of other lncRNAs (HULC, H19, MALAT-1, TUC338, and HOTTIP) that are reported in HCC [20–24]. All of these lncRNAs were up-regulated in HCC (Supplementary Figure S1), which was different from lincRNA-p21. Therefore, we focused on lincRNA-p21 in the present work. The expression of lincRNA-p21 in tumor and adjacent tissues was also analyzed. Markedly, lincRNA-p21 level in tumor tissues was lower than that of adjacent tissues (Figure 1B). We also found the low level of lincRNA-p21 in liver cancer cell lines compared to normal human hepatocytes (Figure 1C). Further, we investigated whether lincRNA-p21 was correlated with clinical markers for HCC. We analyzed the serum levels of alpha-fetoprotein (AFP) in normal donors and HCC patients, and found the high levels of AFP in patients with HCC (Figure 1D). Linear regression analysis implicated the significant but negative correlation between serum AFP level and tissue lincRNA-p21 level (Figure 1E). These data indicate the potential participation of lincRNA-p21 in human hepatocellular carcinoma. To further confirm this correlation, we analyzed whether lincRNA-p21 predicted patients' survival. The results showed that low lincRNA-p21 predicted poor overall and disease-free survival in patients with HCC (Figure 2A and 2B). In addition, we found that low level of lincRNA-p21 was associated with higher tumor grade (*p* = 0.0166) and stage (*p* = 0.0023), and vascular invasion (*p* = 0.0209; Table 1).

**Table 1:** Correlative analysis of lincRNA-p21 levels with clinicopathological features

Characteristic	Number (*N* = 70)	lincRNA-P21 Low (*N* = 38)	lincRNA-P21 High (*N* = 32)	*P* Value
Sex				0.4945
Male	49	25	24	
Female	21	13	18	
Grade				0.0166
1	11	2	9	
2	53	31	22	
3	6	5	1	
Stage				0.0023
1	41	16	25	
2 or 3	29	22	7	
Multiple Tumors				0.5715
No	39	20	19	
Yes	31	18	13	
Vascular Invasion (Macro)				0.1903
No	43	26	17	
Yes	27	12	15	
Vascular Invasion (Micro)				0.0209
No	29	11	18	
Yes	41	27	14	
HBV				0.7434
No	12	6	6	
Yes	58	32	26	
Cirrhosis				0.1613
No	33	15	18	
Yes	37	23	14	

### LincRNA-p21 inhibits hepatocarcinoma cell proliferation and colony formation

**Figure 3:**
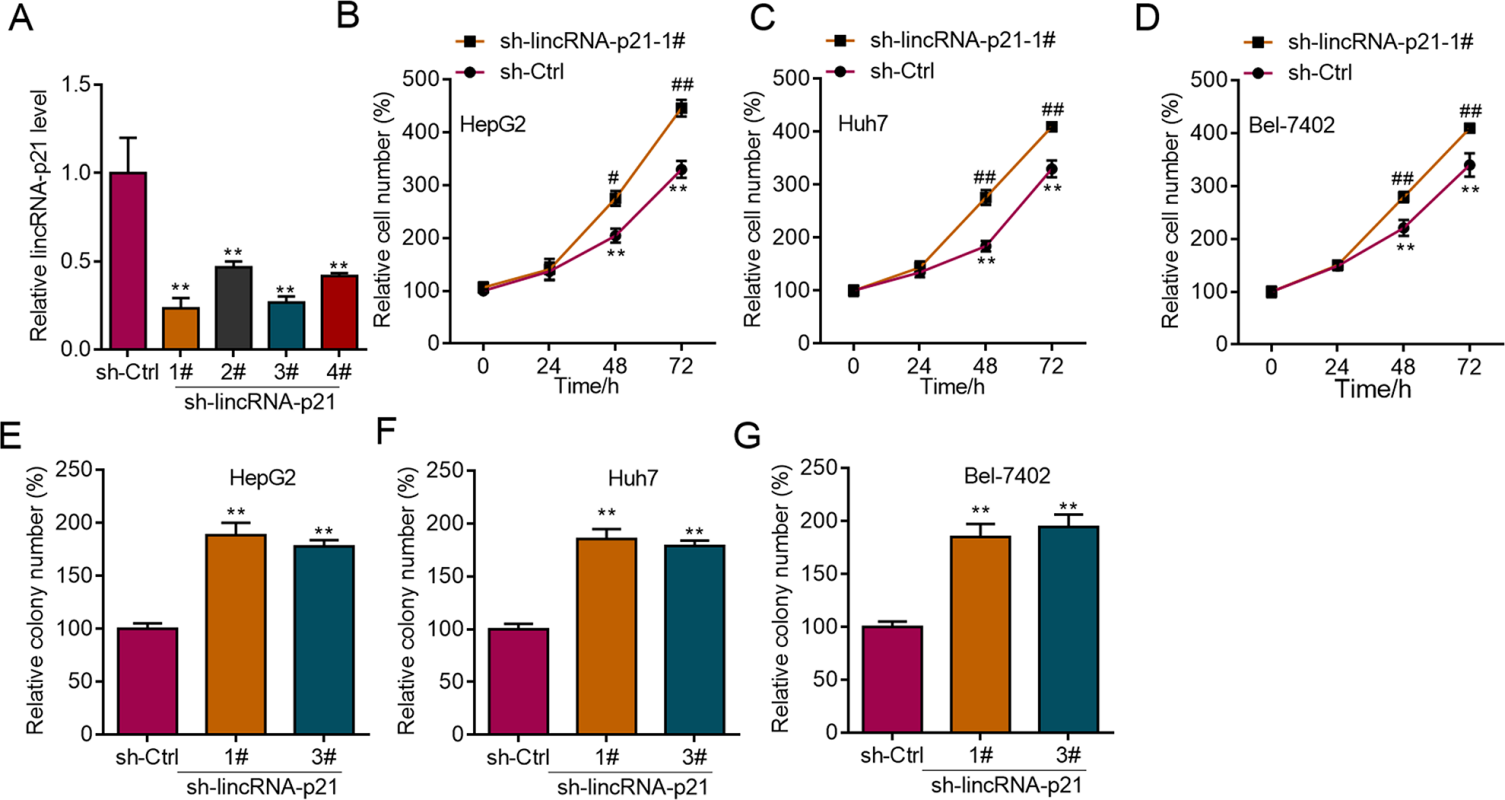
LincRNA-p21 knockdown facilitates proliferation and colony formation of liver cancer cells **A.** Lentivirus-mediated lincRNA-p21 knockdown by short hairpin RNAs (shRNAs) in HepG2 cells. **B–D.** LincRNA-p21 knockdown promotes proliferation of HepG2 (B), Huh7 (C), and Bel-7042 (D) cells. **p < 0.01 *vs*. sh-Ctrl 0 day; #*p* < 0.05 and ##*p* < 0.01 *vs.* sh-Ctrl of the corresponding time points. **E–G.** LincRNA-p21 knockdown promotes colony formation of HepG2 (E), Huh7 (F), and Bel-7042 (G) cells. **p < 0.01 *vs.* sh-Ctrl.

**Figure 4:**
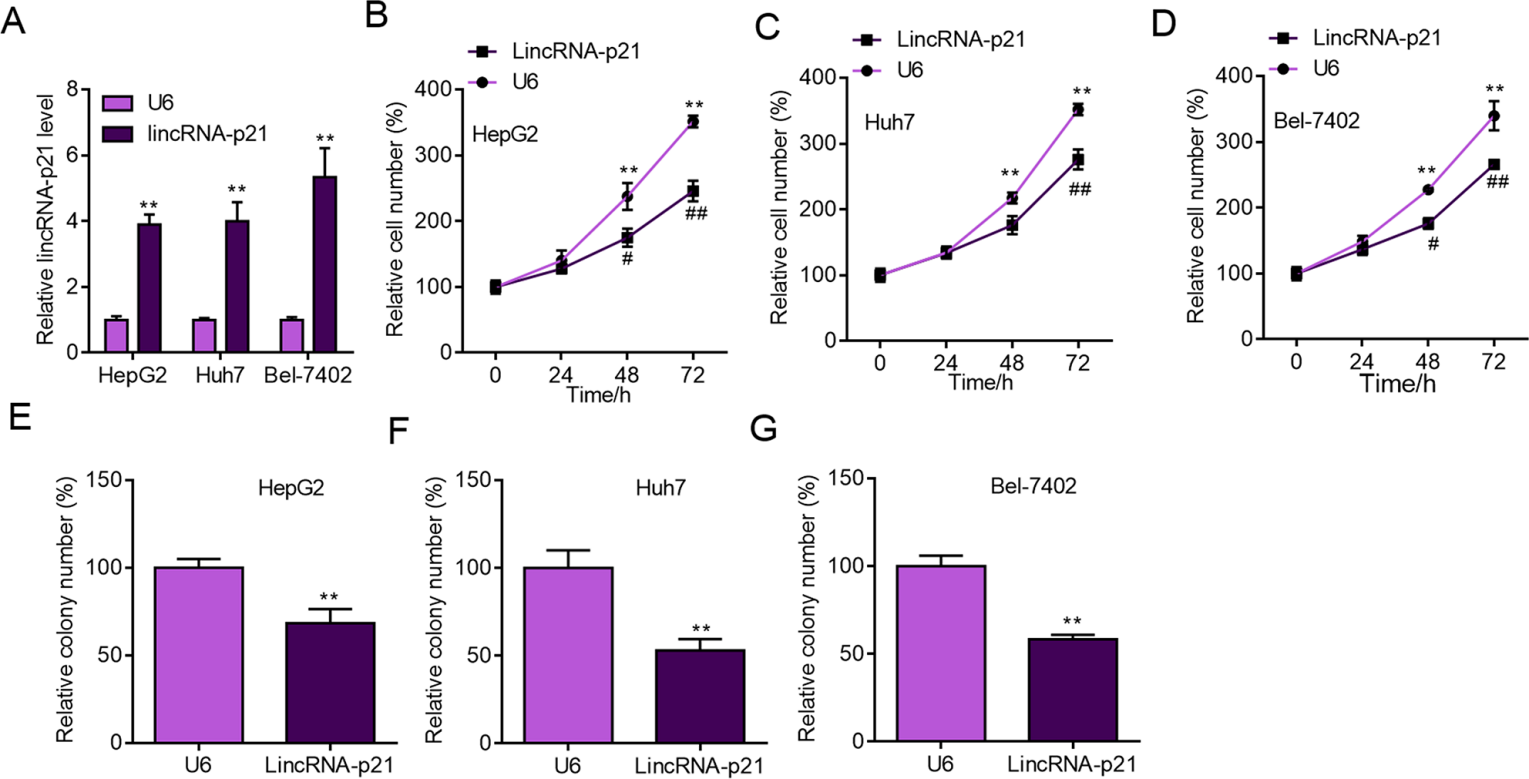
LincRNA-p21 overexpression inhibits liver cancer cell proliferation and colony formation **A.** Lentivirus-mediated lincRNA-p21 overexpression in HepG2, Huh7 and Bel-7402 cells. The level of lincRNA-p21 was checked 24 hours post lentivirus infection. **p < 0.01 vs. U6. $$*p* < 0.01 vs. U6. ##*p* < 0.01 vs. U6. &&*p* < 0.01 vs. U6. **B–D.** LincRNA-p21 overexpression inhibits proliferation of HepG2 (B), Huh7 (C), and Bel-7402 (D) cells. *p < 0.05 and **p < 0.01 vs U6 0 day; #*p* < 0.05 and ##*p* < 0.01 vs U6 of the corresponding time points. **E–G.** LincRNA-p21 overexpression inhibits colony formation of HepG2 (E), Huh7 (F) and Bel-7402 (G) cells. **p < 0.01 vs U6.

The above findings strongly implicated the participation of lincRNA-p21 in human HCC, which prompted us to study the functions of lincRNA-p21 in HCC. We first performed experiments to investigate whether lincRNA-p21 could affect cellular behaviors of liver cancer cells *in vitro*. We knocked down lincRNA-p21 by lentivirus-mediated shRNAs in liver cancer cell lines (Figure 3A and Supplementary Figure S2). Four shRNAs were tested and the first and third shRNAs were selected for further investigation for their relative high knockdown efficiency (Figure 3A). We found that lincRNA-p21 knockdown promoted the proliferation of HepG2 cells (Figure 3B and Supplementary Figure S3A and S3B). LincRNA-p21 knockdown also facilitated proliferation of Huh7 and Bel-7042 cells (Figure 3C and 3D). Further, we investigated whether lincRNA-p21 regulated cellular colony formation of liver cancer cells. The results demonstrated the negative effects of lincRNA-p21 on colony formation of HepG2 (Figure 3E and Supplementary Figure S3C), Huh7 (Figure 3F) and Bel-7042 (Figure 3G) cells. We also overexpressed lincRNA-p21 in liver cancer cells and found that lincRNA-p21 overexpression inhibited proliferation and cellular colony formation of HepG2, Huh7 and Bel-7042 cells (Figure 4A–4G). These findings indicate that lincRNA-p21 inhibits liver cancer cell proliferation and colony formation.

### LincRNA-p21 inhibits liver cancer cell growth and drug resistance *in vivo*

**Figure 5:**
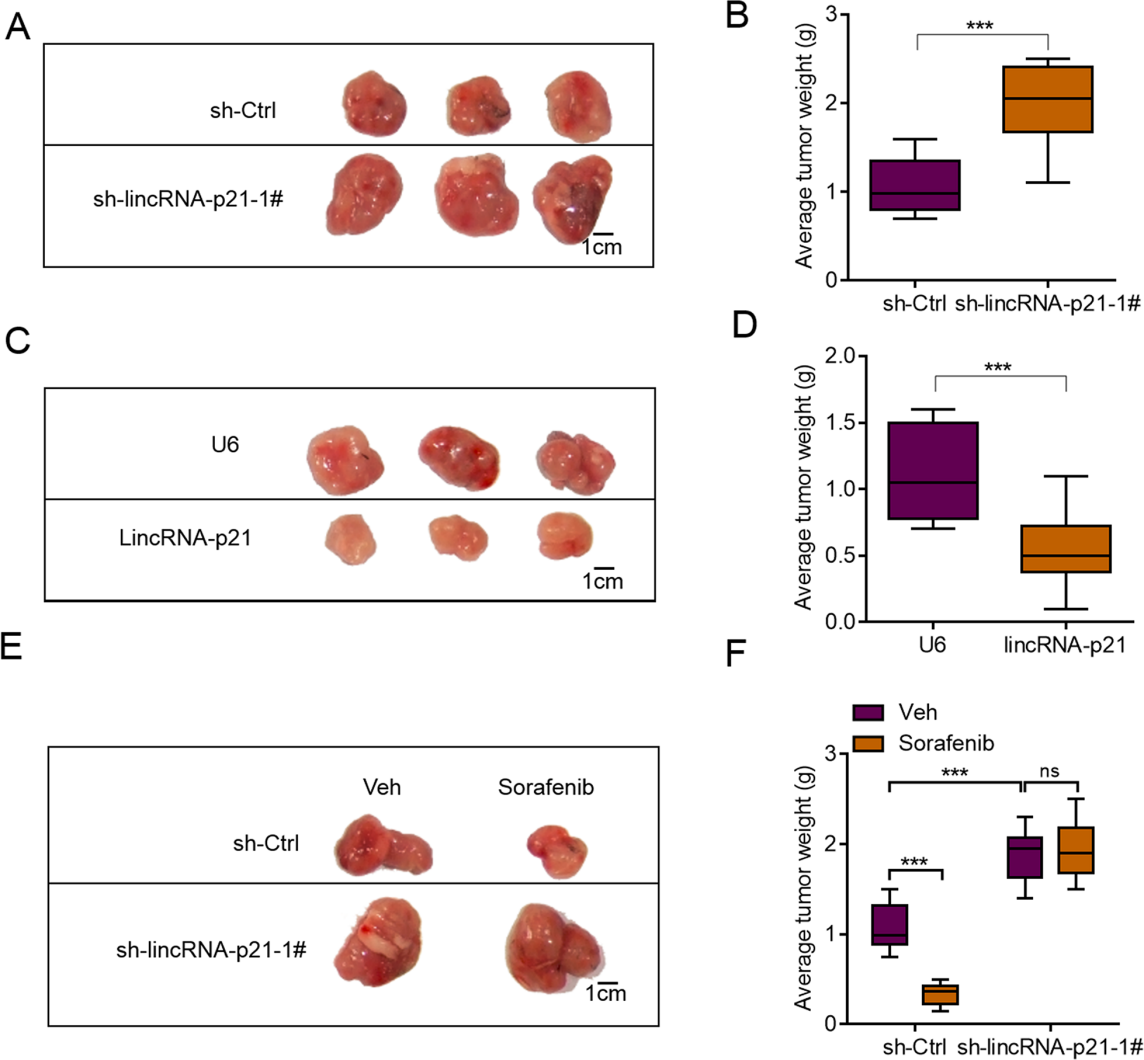
lincRNA-p21 regulates hepatocarcinoma growth *in vivo* **A.** Representative photograph showing tumor size of sh-Ctrl and sh-LincRNA-p21 groups. HepG2 cells were infected with lentivirus-mediated sh-Ctrl or sh-LincRNA-p21 and tumor xenograft experiment was performed. The mice were sacrificed four weeks after tumor implantation. **B.** Quantitative data for tumor weight in sh-Ctrl and sh-lincRNA-p21 groups. *N* = 10 in each group. **C.** Representative photograph showing tumor size of U6 and lincRNA-p21 groups. HepG2 cells were infected with lentivirus overexpressing U6 or human lincRNA-p21 and tumor xenograft experiment was performed. The mice were sacrificed four weeks after tumor implantation. **D.** Quantitative data for tumor weight in U6 and lincRNA-p21 groups. *N* = 10 in each group. **E–F.** LincRNA-p21 mediates sorafenib-induced inhibition of HepG2 cell growth *in vivo*. Sorafenib (30 mg/kg/d) was administered in 100 μL by intraperitoneal injection on day three after tumor implantation. *N* = 10 in each group in (F) ***p < 0.001.

We next studied whether lincRNA-p21 also regulated *in vivo* growth of liver cancer cells. We performed tumor xenograft experiments and knocked down lincRNA-p21. LincRNA-p21 knockdown did not induce any toxicity in mice. We found that lincRNA-p21 knockdown facilitated the *in vivo* growth of HepG2 cells (Figure 5A and 5B). In consistence, lincRNA-p21 overexpression inhibited growth of HepG2 cells *in vivo* (Figure 5C and 5D). These results demonstrated lincRNA-p21 acted as a negative regulator for liver cancer cell growth *in vivo*. Another question we were interested was whether low lincRNA-p21 contributed to drug resistance. Therefore, we used the clinical drug sorafenib, which was reported to induce apoptosis of liver cancer cells partly through inducing ER stress [8, 25]. Firstly, we found that sorafenib could induce the expression of lincRNA-p21 *in vitro* and *in vivo* (Supplementary Figure S4A and S4B). Sorafenib treatment did not induce any toxicity in mice but inhibited *in vivo* growth of HepG2 cells significantly. However, when lincRNA-p21 was knocked down, sorafenib was unable to repress tumor growth (Figure 5E–5F). These findings implicated that lincRNA-p21 downregulation may contribute to clinical tumor growth and drug resistance.

### LincRNA-p21 induces ER stress in hepatocellular carcinoma

**Figure 6:**
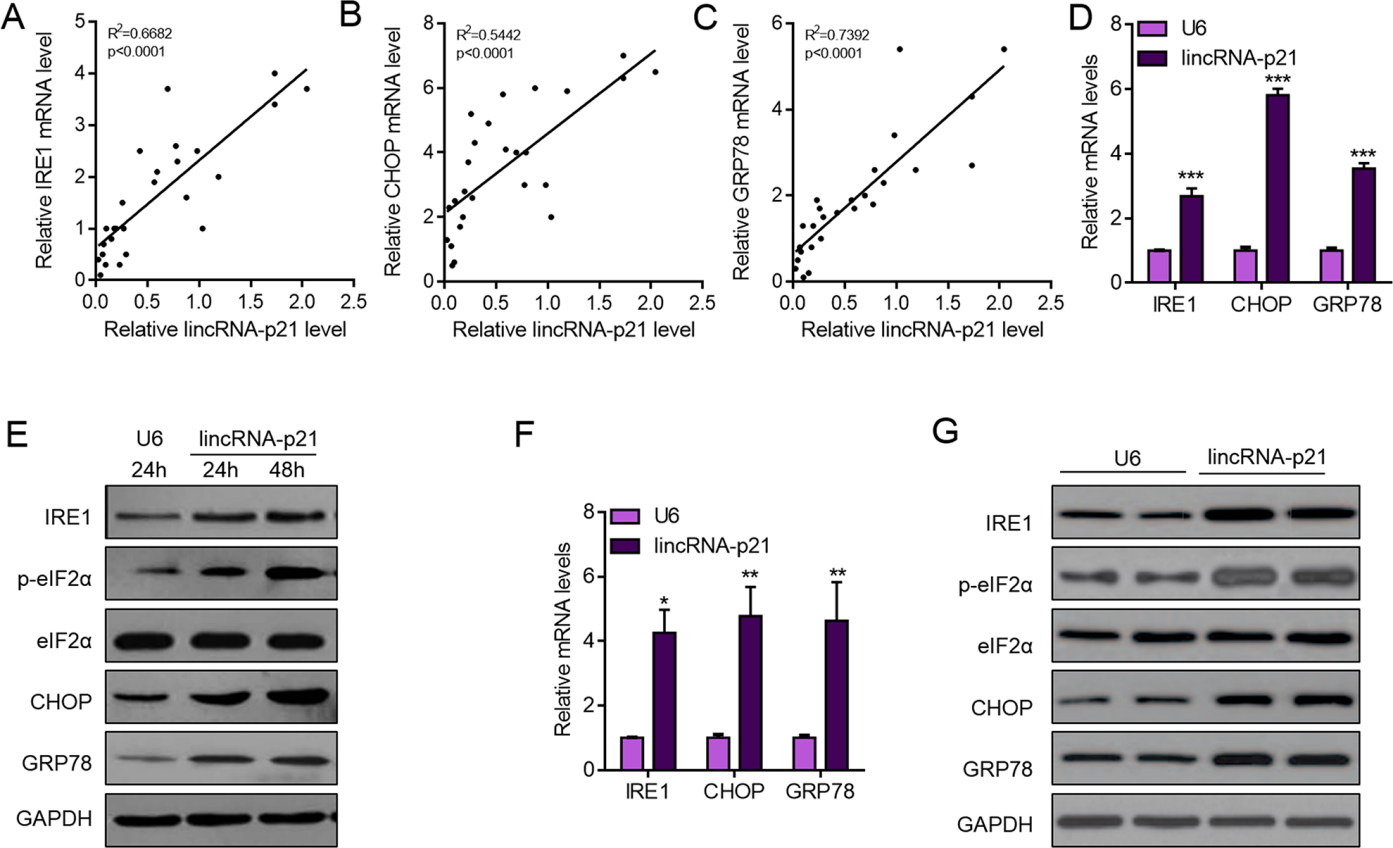
LincRNA-p21 overexpression activates ER stress in hepatocarcinoma **A–C.** LincRNA-p21 level is positively correlated with mRNA levels of ER stress markers (IRE1, CHOP, and GRP78) in human hepatocellular carcinoma. *N* = 26 in each group. **D.** LincRNA-p21 overexpression promotes the mRNA levels of ER stress markers (IRE1, CHOP, and GRP78) in HepG2 cells. HepG2 cells were infected with lentivirus overexpressing U6 or lincRNA-p21 for 48 hours. ***p < 0.001 *vs*. U6. **E.** Western blot showing lincRNA-p21 promotes the activation of ER stress in HepG2 cells. **F.** LincRNA-p21 overexpression facilitates the mRNA levels of ER stress markers *in vivo*. Tumor xenograft experiment was performed using HepG2 cells. The mice were sacrificed four weeks after tumor implantation. *p < 0.05 and **p < 0.01 *vs.* U6. *N* = 5 in each group. **G.** Representative western blot showing lincRNA-p21 promotes the activation of ER stress *in vivo*. Tumor xenograft experiment was performed using HepG2 cells. The mice were sacrificed four weeks after tumor implantation.

**Figure 7:**
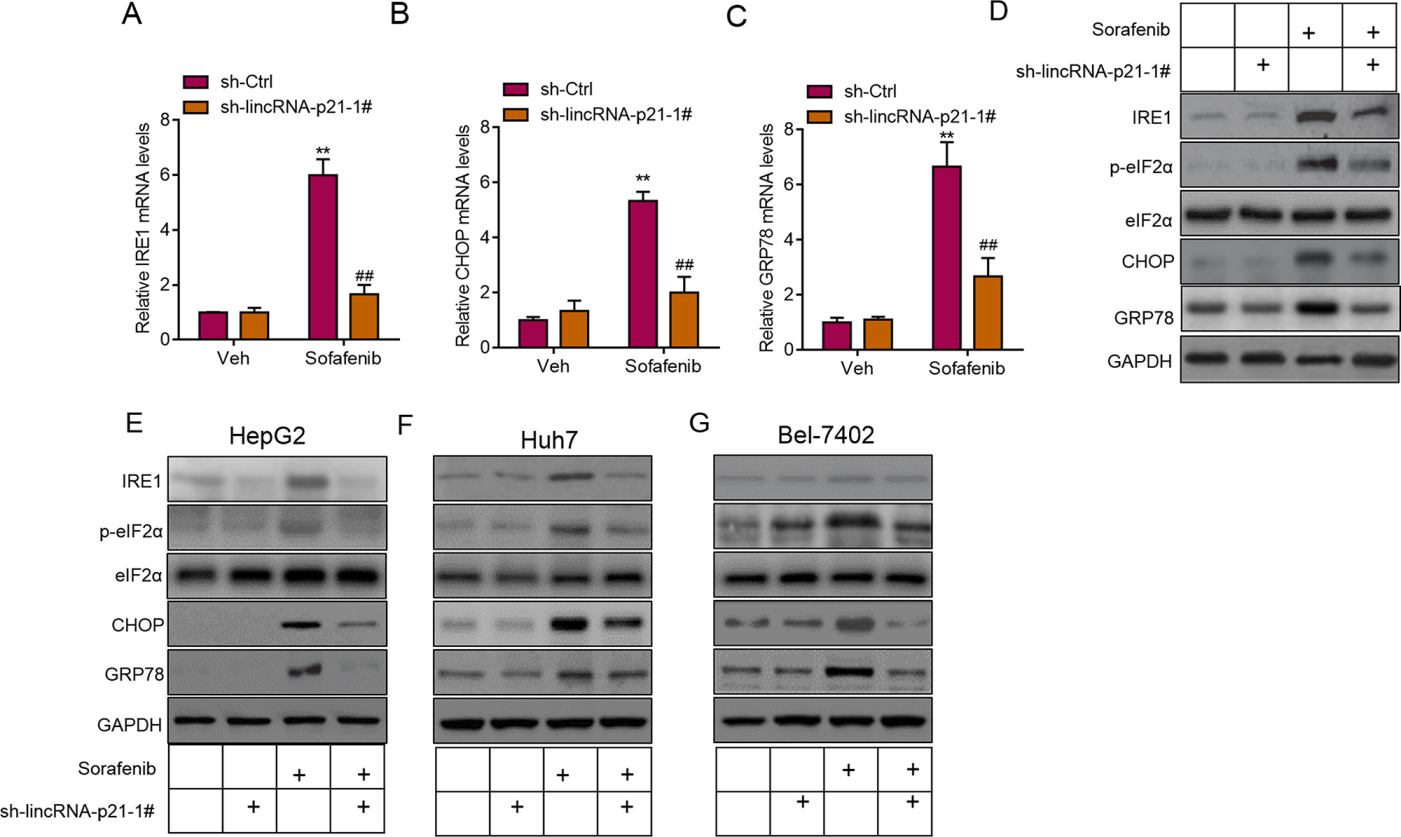
LincRNA-p21 contributes to sorafenib-induced ER stress *in vivo* **A–C.** LincRNA-p21 knockdown inhibits sorafenib-induced expression of ER stress markers *in vivo*. Tumor xenograft experiment was performed using HepG2 cells. The mice were sacrificed four weeks after tumor implantation. Sorafenib (30 mg/kg/d) was administered in 100 μL by intraperitoneal injection on day three after tumor implantation. **p < 0.01 *vs*. sh-Ctrl + Vehicle (Veh); ##*p* < 0.01 *vs*. sh-Ctrl + sorafenib. *N* = 5 in each group. **D.** Representative western blot showing lincRNA-p21 knockdown blocks sorafenib-induced activation of ER stress *in vivo*. Tumor xenograft experiment was performed using HepG2 cells. The mice were sacrificed four weeks after tumor implantation. **E–G.** Representative western blot showing lincRNA-p21 knockdown blocks sorafenib-induced activation of ER stress in HepG2 (E), Huh7 (F), and Bel-7402 (G) cells. Cells were infected with lentivirus carrying sh-lincRNA-p21 or ctrl shRNA for 24 hours followed by 20 μM sorafenib for 48 hours.

Induction of ER stress is one of the important pathways for clinical drugs to induce liver cancer cell apoptosis and repress carcinoma development [8, 10, 11, 26]. Sorafenib is a small molecule that inhibits tumor-cell proliferation and tumor angiogenesis and increases the rate of apoptosis in a wide range of tumor models. In preclinical experiments, sorafenib has anti-proliferative activity in liver-cancer cell lines, and it reduces tumor angiogenesis and tumor-cell signaling and increases tumor-cell apoptosis in a mouse xenograft model of human HCC [27]. One of the major mechanisms by which sorafenib inhibits liver cancer survival is by inducing ER stress [8, 9]. As we showed the contribution of lincRNA-p21 to sorafenib-induced repression of tumor growth, we next wanted to know whether this contribution depended upon ER stress. We analyzed ER stress markers and found the positive correlation between lincRNA-p21 levels and ER stress markers (IRE1, CHOP, and GRP78) in tissues from HCC (Figure 6A–6C). In HepG2 cells, overexpression of lincRNA-p21 induced expression of IRE1, CHOP and GRP78 (Figure 6D). We also found the upregulation of IRE, CHOP and GRP78 protein level and hyper phosphorylation of eIF2α in HepG2 cells overexpressed with lincRNA-p21 (Figure 6E and Supplementary Figure S5). We also tested the activation of protein kinase RNA-like endoplasmic reticulum kinase (PERK), which is responsible for phosphorylation of initiation factor eIF2α, We found that lincRNA-p21 could up-regulate the phosphorylation level of PERK (Supplementary Figure S6). Finally, we studied the effect of lincRNA-p21 on ER stress *in vivo*. In consistent with the *in vitro* findings, lincRNA-p21 overexpression activated ER stress by up-regulating the transcription of ER stress markers (Figure 6F–6G and Supplementary Figure S7). In addition, as reported by previous findings, sorafenib induced ER stress *in vivo* (Figure 7A–7D) and in liver cancer cell lines (Figure 7E–7G). Interestingly, lincRNA-p21 knockdown blocked sorafenib effects on ER stress both *in vivo* (Figure 7A–7D and Supplementary Figure S8A) and *in vitro* (Figure 7E–7G and Supplementary Figure S8B–S8D), indicating that lincRNA-p21 contributes to sorafenib-induced ER stress.

### LincRNA-p21 induces apoptosis of hepatocellular carcinoma cells

**Figure 8:**
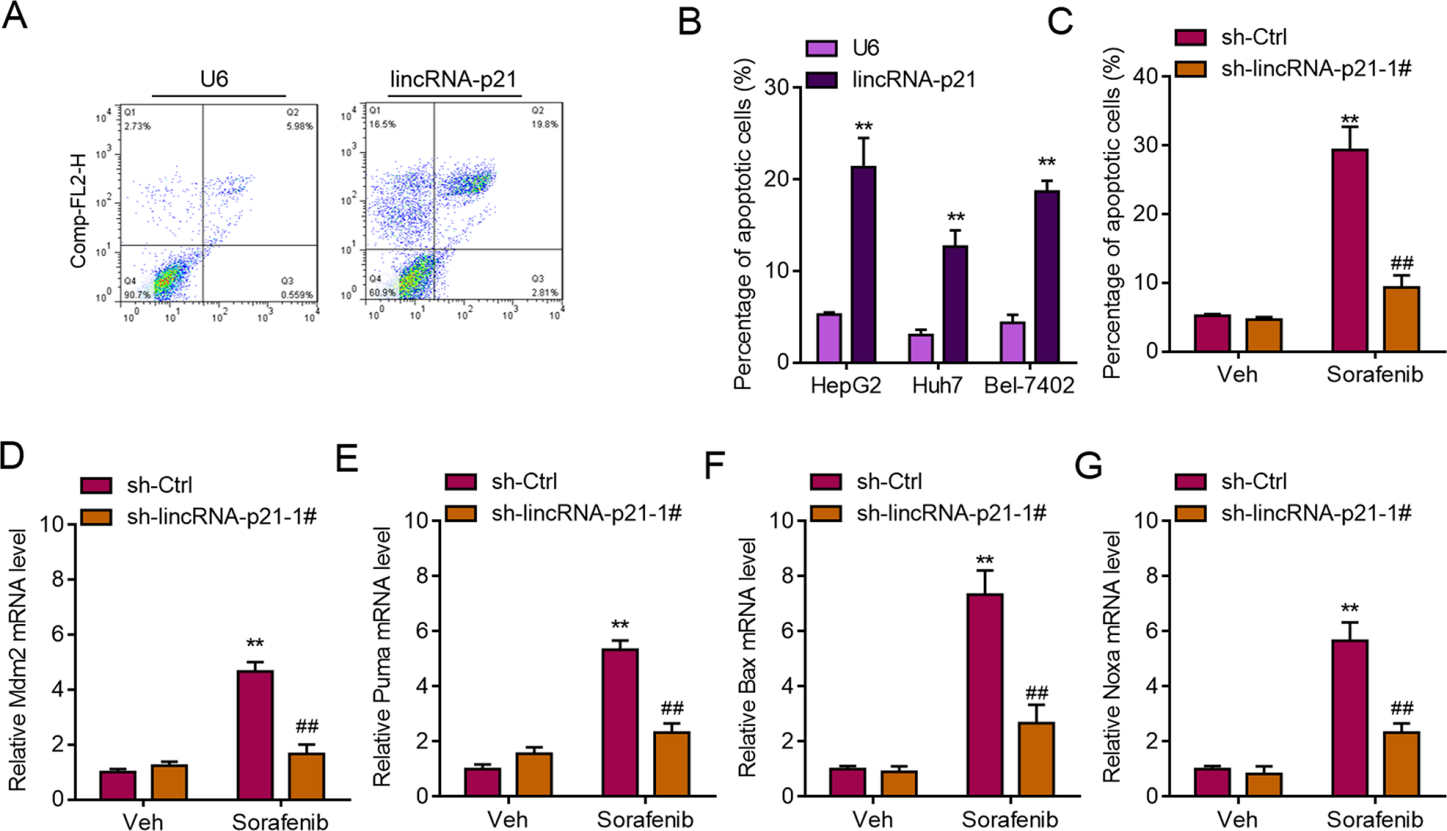
LincRNA-p21 regulates ER stress-related apoptosis **A.** Representative FACS results showing lincRNA-p21 overexpression promotes apoptosis of HepG2 cells. **B.** Quantitative data showing lincRNA-p21 induces apoptosis of HepG2, Huh7, and Bel-7042 cells. Cells were infected with lentivirus overexpressing U6 or lincRNA-p21 for 48 hours. **p < 0.01 *vs*. U6. **C.** LincRNA-p21 knockdown blocks sorafenib-induced apoptosis of HepG2 cells. Cells were infected with lentivirus carrying sh-lincRNA-p21 or ctrl shRNA for 24 hours followed by 20 μM sorafenib for 48 hours. **p < 0.01 *vs*. sh-Ctrl + Vehicle (Veh); ##*p* < 0.01 *vs*. sh-Ctrl+sorafenib. **D–G.** LincRNA-p21 knockdown inhibits expression of sorafenib-induced expression of p53 downstream markers (Mdm2, Puma, Bax and Noxa). Cells were infected with lentivirus carrying sh-lincRNA-p21 or ctrl shRNA for 24 hours followed by 20 μM sorafenib for 48 hours. **p < 0.01 *vs*. sh-Ctrl +Vehicle (Veh); ##*p* < 0.01 *vs*. sh-Ctrl+sorafenib.

High level of ER stress can lead to apoptosis of liver cancer cells [8], therefore, we next studied whether lincRNA-p21 induced HCC cell apoptosis. We found that lincRNA-p21 overexpression induced apoptosis of HepG2 cells (Figure 8A and 8B). Similar results were observed in Huh7 and Bel-7042 cells (Figure 8B). What's more, we showed that lincRNA-p21 knockdown blocked sorafenib-induced apoptosis of HepG2 cells (Figure 8C). As lincRNA-p21 was reported to regulate p53 downstream genes expression [17], we also investigated whether lincRNA-p21 affected these genes expression in hepatocellular carcinoma. We found that lincRNA-p21 knockdown inhibited sorafenib-induced expression of p53 downstream genes, including Bax, Puma, Mdm2, and Noxa (Figure 8D–8G). These findings indicated that lincRNA-p21 contributed to sorafenib-induced apoptosis. ER stress induced apoptosis is mainly mediated through reactive oxygen species (ROS). Therefore, we tested whether lincRNA-p21 regulates cellular ROS level. We found that that lincRNA-p21 overexpression could significantly up-regulated total ROS level (<3.4 fold, Supplementary Figure S9). In addition, lincRNA-p21 also up-regulated the expression of dual oxidase 1 (DUOX1, Supplementary Figure S9), which is the main source of ROS, is significantly decreased in HCC and liver cancer cell lines compared to immortalized normal cell lines and adjacent non-tumor tissues [28]. These results indicated that ROS may be involved in lincRNA-p21-induced ER stress-mediated apoptosis.

### ER stress contributes to lincRNA-p21 effects on hepatocellular carcinoma

**Figure 9:**
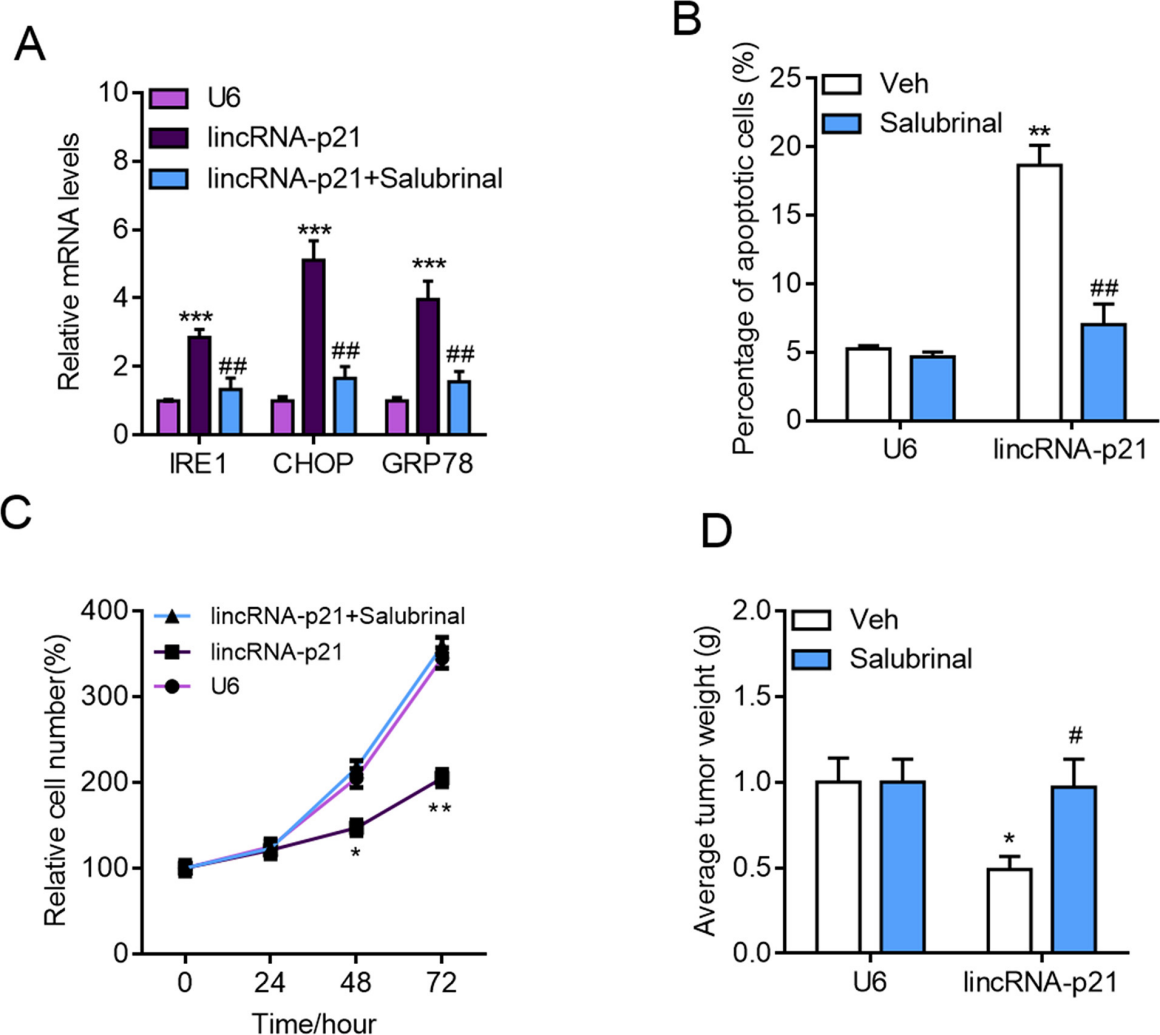
Inhibition of ER stress blocks lincRNA-p21 functions in hepatocarcinoma **A–B.** The cells were infected with indicated lentivirus for 24 hours followed by salubrinal (10 μM) for another 24 hours. (A) Inhibition of ER stress with salubrinal (10 μM) inhibits lincRNA-p21 induced expression of ER stress markers in HepG2 cells. ***p < 0.001 *vs.* U6; ##*p* < 0.01 *vs*. lincRNA-p21. (B) Salubrinal reduces lincRNA-p21-induced apoptosis in HepG2 cells. **p < 0.01 *vs*. U6+Vehicle (Veh); ##*p* < 0.01 *vs*. lincRNA-p21+Vehicle (Veh). **C.** Salubrinal inhibition blocks lincRNA-p21-induced repression of HepG2 cell proliferation. The cells were infected with indicated lentivirus with/without salubrinal (10 μM) for indicated times.*p < 0.05 and **p < 0.01 *vs.* U6 of the same time points. **D.** Salubrinal (1 mg/kg/d) inhibits lincRNA-p21-induced repression of HepG2 cell growth *in vivo*. Tumor xenograft experiment was performed using HepG2 cells. The mice were sacrificed four weeks after tumor implantation. *p < 0.05 *vs*. U6+Vehicle (Veh); #*p* < 0.05 *vs*. lincRNA-p21+Vehicle (Veh). *N* = 10 in each group.

The above results demonstrated that lincRNA-p21 regulated tumor growth, apoptosis and ER stress. One question remained unknown was whether ER stress accounted for lincRNA-p21-induced apoptosis and growth arrest. Therefore, we inhibited ER stress with salubrinal, a reported inhibitor for phosphorylation of eIF2α, in HepG2 cells (Figure 9A). Salubrinal treatment significantly rescued lincRNA-p21-induced apoptosis of HepG2 cells (Figure 9B). Significantly, salubrinal also inhibited lincRNA-p21-mediated growth inhibition of HepG2 cells *in vitro* and *in vivo* (Figure 9C and 9D). These findings demonstrated that ER stress, at least in part, contributes to lincRNA-p21-induced apoptosis and growth arrest.

## DISCUSSION

Using *in vitro* and *in vivo* evidence, we demonstrate here that lincRNA-p21 serves as a suppressor for HCC development and drug resistance partly by activating ER stress. We first show the downregulation of lincRNA-p21 in patients with HCC, which is correlated with higher disease stage and poor survival. And then we demonstrate that lincRNA-p21 inhibits growth of liver cancer cells *in vitro* and *in vivo*. Our mechanism analysis reveals that lincRNA-p21 activates ER stress and mediates sorafenib-induced ER stress, apoptosis and growth arrest. Finally, we provide evidence that lincRNA-p21 effects on liver cancer apoptosis and growth partly depends upon induction of ER stress.

LincRNA-p21 is directly induced by p53 to play a critical role in the p53 transcriptional responses [15]. Both protein and mRNA targets for lincRNA-p21 have been identified [15, 29]. Previous reports showed the involvement of lincRNA-p21 in cell cycle [19], Warburg effect [16], and cell survival [17], and these properties implicating functional roles of lincRNA-p21 in carcinoma. Chou *et al*. [30] reported the involvement of lincRNA-p21 in mammary cancer cells through control of HuR/elavL1 expression. In colon cancer tissues, lincRNA-p21 is significantly down-regulated [31, 32]. In human HCC, lincRNA-p21 level was also down-regulated. The expression level of lincRNA-p21 was negatively correlated with AFP serum level, and low lincRNA-p21 level predicted poor overall and disease-free survival. Therefore, lincRNA-p21 could serve as a prognostic factor for favorable outcome. We used three HCC cell lines to study the function of lincRNA-p21 *in vitro* and *in vivo*. Both loss-of-function and gain-of-function experiments provided evidence that lincRNA-p21 was a tumor suppressor lincRNA, and treatments that activate lincRNA-p21 may be promising for HCC therapy.

Here we identified a new biological process that lincRNA-p21 participates in. We found that lincRNA-p21 could activate ER stress by increasing the expression of IRE1, CHOP and GRP78. However, in the reported targets of lincRNA-p21, none of these genes were observed [15], indicating that lincRNA-p21 may regulate ER stress indirectly. These finding identified new biological function of lincRNA-p21 other than metabolism, and cell reprogramming [15, 16, 19, 29]. However, how lincRNA-p21 regulates ER stress remains to be further explained.

Sorafenib is a clinical drug for HCC [25]. Previous reports indicated that sorafenib induce apoptosis and growth arrest of HCC cells partly through activating ER stress [5–9]. However, the underlying mechanism remains largely unexplored. We identified here lincRNA-p21 contributed to sorafenib-induced ER stress and apoptosis. Knockdown of lincRNA-p21 blocked sorafenib effects on ER stress, apoptosis and growth inhibition, indicating lincRNA-p21 low expression is one of the reasons for drug resistance in HCC and high level of lincRNA-p21 may predict favorable outcome post sorafenib therapy. We also found that sorafenib induced expression of p53 downstream genes, Puma, Mdm2, Noxa and Bax, which was blocked by lincRNA-p21 knockdown. Therefore, lincRNA-p21 may also target p53 to induce apoptosis as previously reported [17]. Previous reports show that lincRNA-p21 increases following X-ray treatment, and enforced expression of the lincRNA enhances the sensitivity of radiotherapy for CRC by promoting cell apoptosis [32]. These findings implicate that up-regulation of lincRNA-p21 may contribute to drug treatment and radiotherapy.

In conclusion, we identify lincRNA-p21 as a suppressor for HCC growth and drug resistance through ER stress, and further explain how sorafenib regulates ER stress and HCC development. LincRNA-p21 may serve as a prognostic factor for outcome and drug resistance.

## MATERIALS AND METHODS

### Patients and tissue specimens

70 cases of hepatocellular carcinoma (HCC) patients with full case history between May 2001 and October 2007 were enrolled in the present study. Fresh HCC or adjacent normal liver tissues were obtained and stored at −80°C before use. The patients were recruited at Eastern Hepatobiliary Surgery Hospital, Second Military Medical University (Shanghai, China). All HCC samples were histopathologically re-evaluated independently by two pathologists before further analysis. Healthy liver samples were obtained from donor livers used for transplantation that were pathologically evaluated before transplantation. Further patient information is included in Table 1. A written form of informed consent was obtained from all patients and donors before any treatment and experiments. The study was approved by the Clinical Research Ethics Committee of Second Military Medical University. The methods were carried out in accordance with the approved guidelines.

### Quantitative real-time PCR (q-PCR)

Total RNA was extracted from cells or fresh tissues with TRIzol (Invitrogen # 10296010). cDNA was synthesized from two μg of total RNA with One-Step RT-PCR Kit (TaKaRa # RR086A). Then, q-PCR was performed with the SYBR Green (TaKaRa # DRR420A) on an ABI-7500 RT-PCR system (Applied Biosystems). The primers were listed in Supplemental Table S1.

### Cell culture and retroviral transduction

The liver cancer cell lines HepG2, Huh7, Hep3B, LM9, Bel-7402 and SMMC-7721 were cultured as described previously [33]. Normal human hepatocytes were isolated from specimens obtained from patients undergoing hepatic resections for the therapy of hepatic tumors after informed consent and according to the rules of the ethics committee of the Second Military Medical University. The liver cancer cells and normal human hepatocytes were cultured in high glucose-containing Dulbecco's modified Eagle's medium (DMEM) supplemented with 10% FBS, 100 units/ml penicillin and 100 μg/ml streptomycin.

Sh-lincRNA-p21 and control shRNA (sh-Ctrl) lentivirus particles were purchased from GenePharma. The shRNA sequences targeting lincRNA-p21 is shown in Supplementary Table S2. Lentivirus expressing human lincRNA-p21 was generated by sub-cloning mouse lincRNA-p21 cDNA to the pSLIK lentivirus expression system (cloning primer forward: TGGCAGTCTGACCCACACTCCCCACGCCC; reverse: ACAGTGCACAGACAATCATACACACGTGT). For retroviral packaging, 293T cells were co-transfected with the retroviral particles. For transduction, cells were incubated with virus-containing supernatant in the presence of 8 mg/ml polybrene. After 48 hours, infected cells were selected for 72 hours with puromycin (2 mg/ml) or hygromycin (200 mg/ml).

### Cell proliferation assay

Cell proliferation ability of the liver cancer cells was monitored by CCK-8 Cell Proliferation/Viability Assay Kit (Sigma # 96992) in according to the guidelines.

### Colony formation

For liver cancer cell colony formation assay, liver cancer cells were suspended in 1.5 ml complete medium supplemented with 0.45% low melting point agarose (Invitrogen # 16520-050). The cells were placed in 35 mm tissue culture plates containing 1.5 ml complete medium and agarose (0.75%) on the bottom layer. The cells were cultured for 14 days. Cell colonies were stained with 0.005% crystal violet and analyzed using a microscope. The colony number in each well was calculated.

### Tumor xenograft experiments

Equal numbers of HepG2 cells stably expressing either control or lincRNA-p21 knockdown, U6 or lincRNA-p21 overexpression vectors (5 × 10^6^) in 100 μl of a 1:1 mixture of culture medium and growth factor–reduced Matrigel were implanted subcutaneously into the forelegs of 4- to 5-week-old male BALB/c athymic nu/nu mice (Vital River). When the tumors reached approximately 7–10 mm in diameter, they were prepared to form a brei and then injected subcutaneously into nude mice. Tumor growth was monitored by tumor weight at the end of study (Four weeks for *in vivo* tumor growth). The study was approved by the Animal Research Ethics Committee of Second Military Medical University. The methods were carried out in accordance with the approved guidelines.

### Western blot

Cells or fresh tissues were lysed in RIPA lysis buffer with mixture of protease inhibitors (Beyotime #ST506) and Phos*STOP* (Roche #04906845001). 30 μg total proteins were subjected to 12% SDS–polyacrylamide gel. After electrophoresis, the proteins were transferred to PVDF membranes, which were then blocked with 5% milk for 2 hours. The membranes were then probed with primary antibody for IRE1 (Abcam # ab37073), CHOP (Cell Signaling Technology # 2895), GAPDH (Santa Cruz #sc32233), GRP78 (Abcam # ab32618), p-eIF2α (Cell Signaling Technology # 9721), eIF2α (Cell Signaling Technology # 3597), DUOX1 (Abcam #ab78919), p-PERK (Cell Signaling Technology #3179), PERK (Cell Signaling Technology #3192) at 4°C overnight, and then the membranes were washed with TBST and incubated with HRP-conjugated secondary antibodies (Santa Cruz # sc-2030) for 1.5 hours and finally washed and visualized using Chemiluminescent ECL reagent (Beyotime # P0018).

### Apoptosis assay

Apoptosis of liver cancer cells was evaluated with fluorescence-activated cell sorting (FACS) assay. FACS analysis was conducted with an Annexin V-FITC Apoptosis Detection Kit (Abcam # ab14086) according to the manufacturer's protocol. A FACS Calibur flow cytometer was used for data analysis.

### ROS determination

Dihydroethidium (DHE, Invitrogen #D11347) was used for detecting ROS generation in cardiomyocytes as per the manufacturer's protocol.

### Statistics

Values were expressed as Mean ± SEM. Statistical differences between two groups were determined using unpaired or paired Student's *t* test. For more than two groups, one-way or two-way ANOVA analysis were applied. The correlation of lincRNA-p21 levels with patients' clinicopathological variables was analyzed by the χ2 test or Fisher's exact test. The Kaplan-Meier method was used to estimate overall and disease-free survival. Survival differences according to lincRNA-p21 expression were analyzed by the log-rank test. Linear regression analysis was performed to analyze the relation between lincRNA-p21 level and AFP serum level as well as ER stress markers in patients with HCC. *P* values of less than 0.05 were considered statistically significant.

## SUPPLEMENTARY INFORMATION FIGURES AND TABLES


