# Supplementary material for: LincRNA-p21 activates endoplasmic reticulum stress and inhibits hepatocellular carcinoma
Source: Oncotarget. 2015 Jul 20;6(29):28151–63. doi: 10.18632/oncotarget.4661 (PMC4695050; doi:10.18632/oncotarget.4661)
Supplement: Supplementary file 1 [file oncotarget-06-28151-s001.pdf]

SUPPLEMENTARY INFORMATION

HUMAN LINCRNA-P21 SEQUENCE

> lincRNA-p21

TGGCAGTCTGACCCACACTCCCCACGCCCA
GGACCAAGTCGCCTGAGCCCCATAGCCACAACT
CTCTGCCGGCCTTGCCCGGGCTTGCCTTGGTTGC
ATCATCTCCCAGCTTTGCCAGGGGTGCAGAAGTG
AACCACCCACTCAGCGCTGGAAAAACCAGCTAA
TTATATCTCCAAAGACCCAGGGCAAGAACTTGTG
GACAACCTCAGCTGGCCTGGCCTGTCCCACTCGC
TTTCCATTTCCCCCCACCCTGAGACAGGATGCCA
CTGTGTAGCCCAGTGTAGTGAACACTTGGAATAC
AAAATAAAGATGGTGGAATGAGACATTCCGTCTC
CAGTTCCTAACATCAGAAGTGAGCACCTGTGTGT
CACCAGCAACATATTGGAGGCCAGCTGCCTACAC
CAATCAGAAACAGGGACCACAAAGTCTGCAGGG
GTGAGATAGGCCTTTTCAGTGTCTACGATTTCATC
ATGGACATTACTGTCGATTTCTCTTCCTGCCCCTG
TATGTGCGGGTCTATCGCCATCTCGCCATCTCCGG
CTCCTGTGTTATGAAGACAGTCTCATGCAGCCCA
GACCAATCTCAAATTGACTAGGTAAACTGAGGCT
GGCCTTGAACCAGTCCCTCCTGCCTCTATCTCCTG
AGAGCTGGGTAACAGCTTCTGGAGCCTCACCCA
GCTTCGCTTTCACCACCCCAGCCAATCCTGTGAC
TCCTCTTCATAGCGAGAGCATTGACACTTATGTTC
TGAGTGTGAAAAAAAAAAAAAAAATCACATAGTA
CTTCCTACTAGAACCTTGCCCGGGTCAACTGAAG
TGTGTGTGTTTGCTTACCAGTCTGCAGGTTTGCTT
AAGTTTGTTTATTTTAGAGACCAGGTCTTGCTCTG
TTGCCTAAGCCAACTTGAAACTCTTGGGCTCCAA
CAGTTCTCCTGCTCCATCTTTCTAGAGCAGCTGG
GACTCTAGGCATGCTCCACTACAGCTGACTGAAG
CTTGAATGAATGAATGAATGAATGAATGAGTTAC
TACCTCTGAGGGACCCTCCTCATGGCCTTCTCAA
CTCTGTACTTTAATTAGTTGCTTAGAGGTGTCTCC
ATGCCTCAGTTTCCCCATCTGTAATAGTAAACGA
GTTCACAGGCGTGGTCTGTGCTTGGAGCAAGAA
CAAAACCATCCTGGCCTATACTCTCTAGGGACTC
TACAGAGCCCATCCCTTTGGCTATCAGACTGTTG
AGATAAAGGTTCCCCCAGGAATCGGAATCCCCTC
CGACAGGAGTCTCATGCTCAGAGAAGAAAAAAA
GCTGAAGCCTGCTGACAGCCAGAGAGGGTACTT
GTCTGTCAGGGAAAAATTCTACACAGGACAGAC
TGGAGCCCAGACTGGTCTGGGTCAGTGACACAC
GGCACAGCACACGGTGGGACAACCTGGCTGTGC
ATCTCTCTCAAACCTCTTGTCACCCTTCTCACTG
TGGCCACAGCGAAGCGAATACATCTCTCTGCTTT
TCACTACGTAGCTCCATCATTGCCTTGGGGGGG
GTAGCGAGGAAGGTCACTGGGGCCCTGCCTCTG
ATAAGAAGAACGAGCAATTATGATTTCCAGGAA
CCGAGGGTGCTCTTGCTGTTCAGTGTCTCCAGCA
CCCCCGGAGACCCCAGGGCTGCCGTCAAGGGTG
TTCAATAAACACGTATCGATTGAGCCAACAATGC
CAGAATTGGACCTGCAGAGGAGAAAATGGACAA
ACAAGACAGTGAGCCTGCAGGTGAGACCAGAA
CTGGAGCCAACAATCTACCTCTCTCTCCCAACCC
TAGCAACGCCAGCAGCTCTCTGGGCGAGGGGCA
CAGTTGCTTCCAGTTGGCAGAACCAGTCTCCCA
GCTTCCCTTCAGAACCCAGCACCTGCTGAGCCA
CCGACCCACGGACTGTCTCTCTCTGGAAGGCAG
CCACCGACCCACGGACTGTCTCTCTCTGGAAGG
CAGTTCACCTCTGGGTTTCAACACTGCCCTTTCC
CCTTTCCTTCTTTAGCCCTTAGGAATCCCTGAAA
GCTTCCTGTGCTTGTGGCTTCTGTGACTTCTCAA
CATCTCTTGTGCACACACACACACACACACACA
CACACACACCAGCCTGTGTCTAAGCAGTTCATC
CTGTACAATGTCTCTCTGATAAAATAACTGATTC
CATTTCTGTCACCTGCTGAGGCTCCAGCAGCTCT
GCCCTCAGACCTCTTAAAGCTGAGGCTCAGAAG
CACAGGGAGGCACGGGAACCTGGTCCCAGGCC
CTGGCTTGCTGGAGCGAAGGAATCTATTGCTTCG
GCCACTCGGTCAGAATCCCTCGGAGATTGATGTG
ATATGCACAGTGACACCCAATCGGGCTTGGAAAA
CTGGGCCAACAGTTAAGCCACACAAAGGAACTA
ACCACAGCTCCACTGGCAACTGGCTCCTTGGCA
AGTGCCAAAACAAACAGCTGTGGTGCAGGCCTT
CCCCGGGCTGCCGGCTTCCTGGACACTGGCAGA
GGCCGCTCAAGAAGGGAGTACCTGAGTAGGGTG
TTGTTCAGTTGGTAGAACGTTTGCTTGCCTTCCA
TGAAGCCCAGGGTTCTGTCTGCACCTCATACCTG
TGATCCTAGCAGTTGGGAAAAGACAGCAAGCAC
CCGGATCAGAAGTTCAAGACCACCCTCCCCTTTA
TAAAGGGATCTGAGGCAGCCTGGGACATCTGAA
TGACAAATGAAAAGAGCCGTGAGCTATCTGGTG
TTTTCTTCATGGAAGTCCAAGTCTCCCCCTCATT
CCTCCCAGGATTCTCCGAATCTGGCTGTTGTCTT
TTGCGATATTTTAGAATATTCTAGCCAGAGCGCA
GAGTATAAAATACAAGTCAAAGGCAATGAGCATA
TGTTAGATGGATGGAGGAGGGCCTAAAAATGCT
GGTGCTGGGGAGGAAAAATGGCTCAGCAGTTAA
GAGCACCGGCTGCTTTTACAGAGGACCTGCCTT
CAGTTCACAGCAACCGCATGACGGCAACTCTGG
TTCCAGGAAATCCAATGCCCTCTTCTGACCTCTA
TAGGCACCCGGCAAGCACACAGTGCACAGACAA
TCATACACACGTGT.

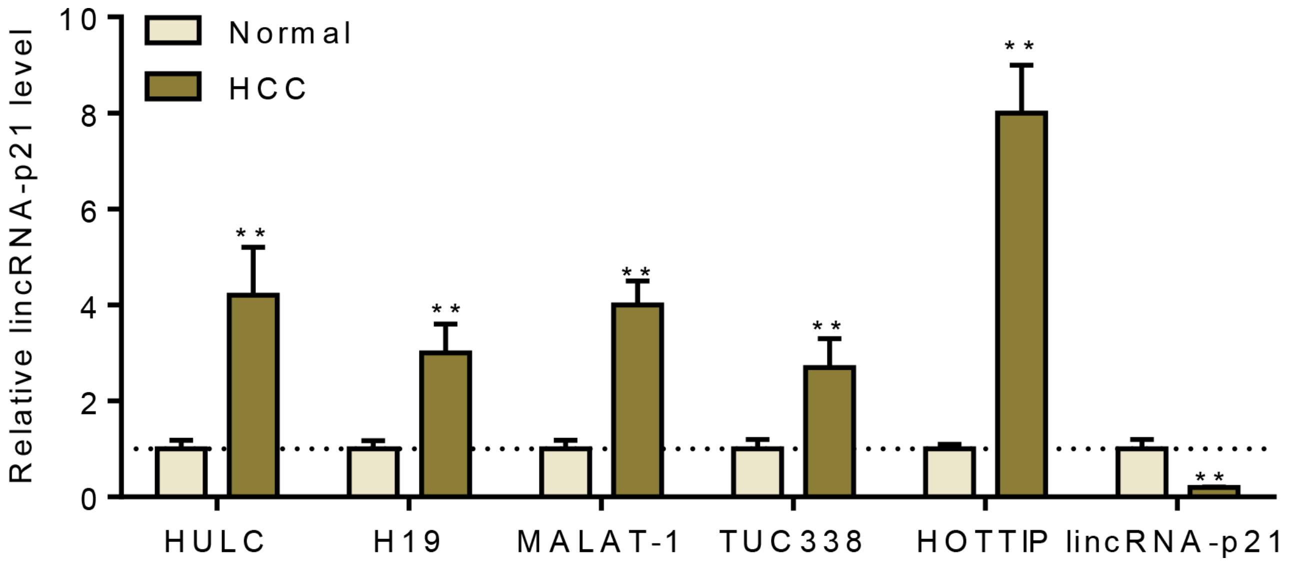

Supplementary Figure S1: lncRNAs level in normal liver tissues and human hepatocellular carcinoma (HCC). **$p < 0.01$ vs. normal. $N = 10$.

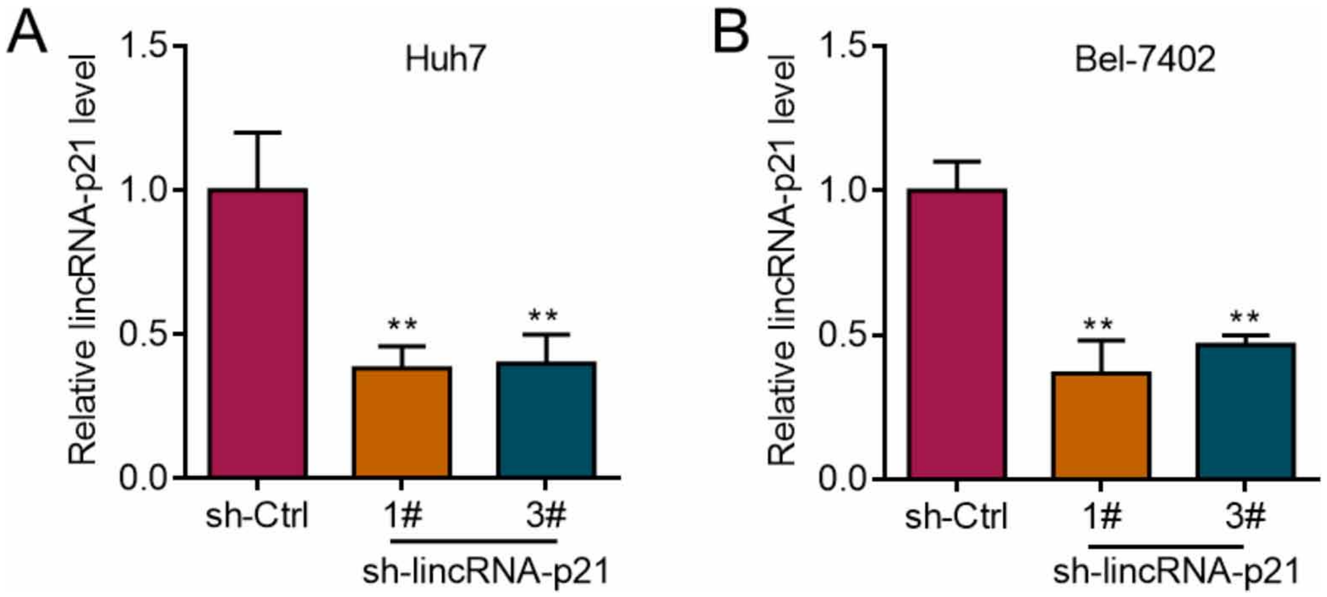

Supplementary Figure S2: Lentivirus-mediated lincRNA-p21 knockdown in Huh7 and Bel-7402 cells. Huh7 **A.** and Bel-7402 **B.** cells were infected with lentivirus carrying sh-Ctrl or sh-lincRNA-p21 for 48 hours. The level of lincRNA-p21 was analyzed with q-PCR. ***p* < 0.01 vs. sh-Ctrl.

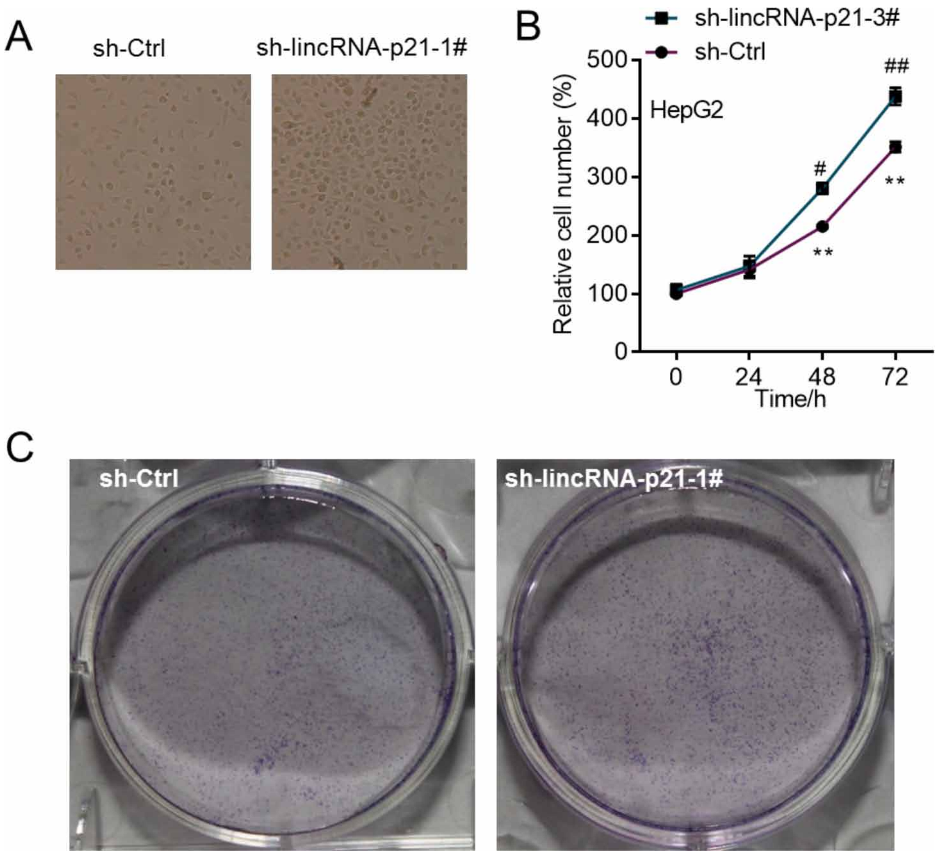

Supplementary Figure S3: LincRNA-p21 knockdown promotes cell proliferation and colony formation in HepG2 cells. **A.** Representative image showing lincRNA-p21 knockdown promotes HepG2 cell proliferation. The photographs were captured 48 hours post infection of lentivirus. **B.** LincRNA-p21 knockdown promotes HepG2 cell proliferation. $^{**}p < 0.01$ vs sh-Ctrl 0 day; $\#p < 0.05$ and $\#\#p < 0.01$ vs sh-Ctrl of the corresponding time points. **C.** Representative photograph showing lincRNA-p21 knockdown promotes colony formation of HepG2 cells.

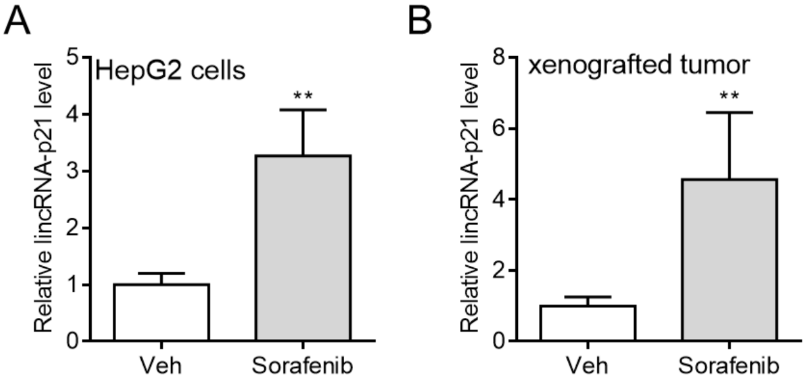

Supplementary Figure S4: Sorafenib promotes lincRNA-p21 expression. **A.** HepG2 cells were treated with 20 μM sorafenib for 48 hours. LincRNA-p21 expression was analyzed with q-PCR. $^{**}p < 0.01$ vs control (Veh). **B.** Tumor xenograft experiment was performed. The mice were sacrificed four weeks after tumor implantation. Sorafenib (30 mg/kg/d) was administered in 100 μL by intraperitoneal injection on day three after tumor implantation. LincRNA-p21 expression was analyzed with q-PCR. $^{**}p < 0.01$ vs control (Veh). $N = 5$ in each groups.

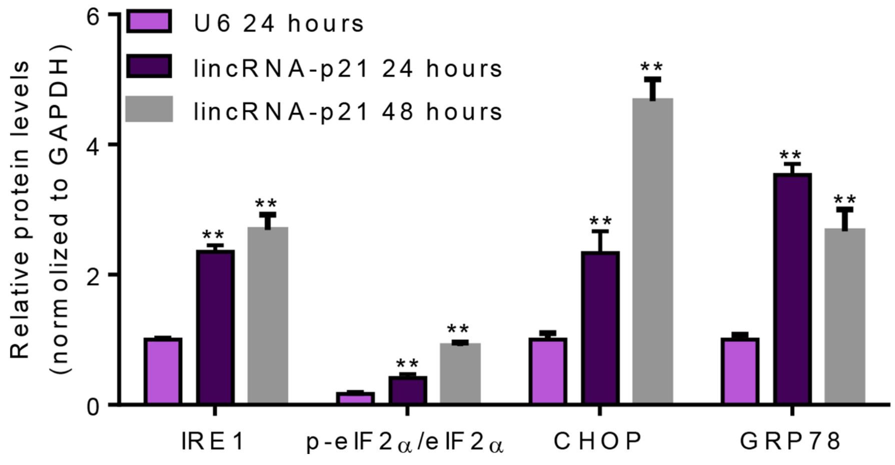

Supplementary Figure S5: Quantitative data related to Figure 6E. **$p < 0.01$vs. U6 24 hours.

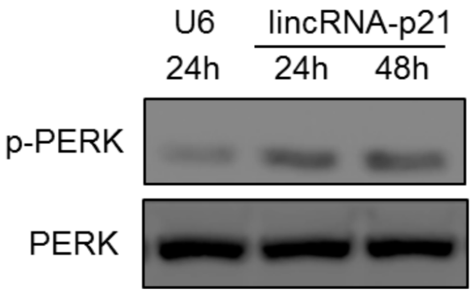

Supplementary Figure S6: lincRNA-p21 activates PERK (Protein kinase RNA-like endoplasmic reticulum kinase). HepG2 cells were infected with indicated lentivirus for indicated times.

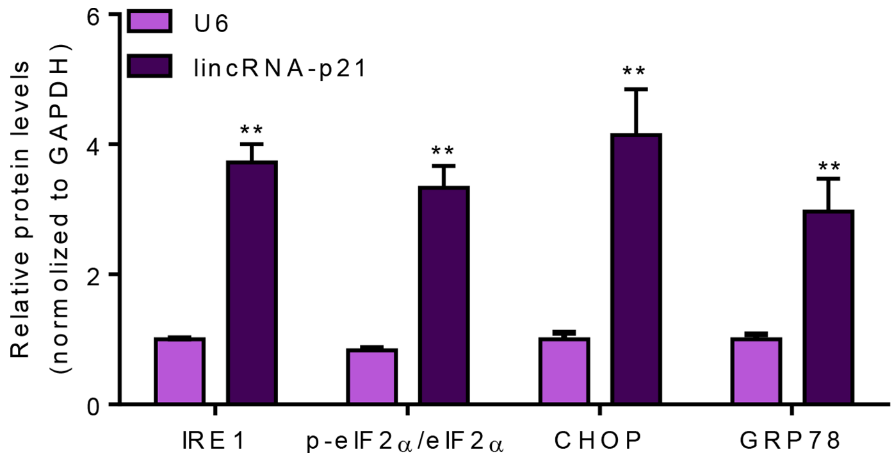

Supplementary Figure S7: Quantitative data related to Figure 6G. **$p < 0.01$ vs. U6 24 hours.

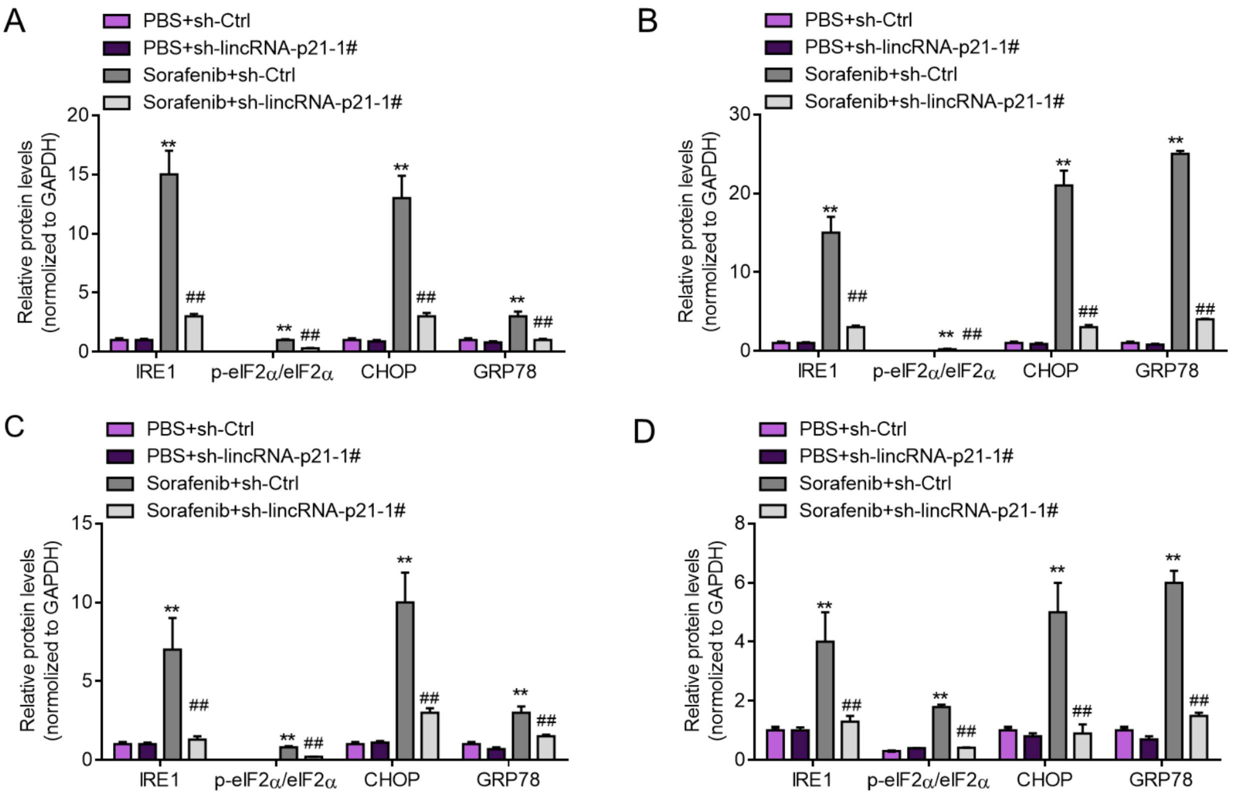

Supplementary Figure S8: Quantitative data related to Figure 7D–7G. A. Quantitative data related to Figure 7D. $n = 5$. **B.** Quantitative data related to Figure 7E. The experiments were repeated for three times. **C.** Quantitative data related to Figure 7F. The experiments were repeated for three times. **D.** Quantitative data related to Figure 7G. The experiments were repeated for three times. ** $p < 0.01$ vs. PBS+sh-Ctrl; ## $p < 0.01$ vs. Sorafenib +sh-Ctrl.

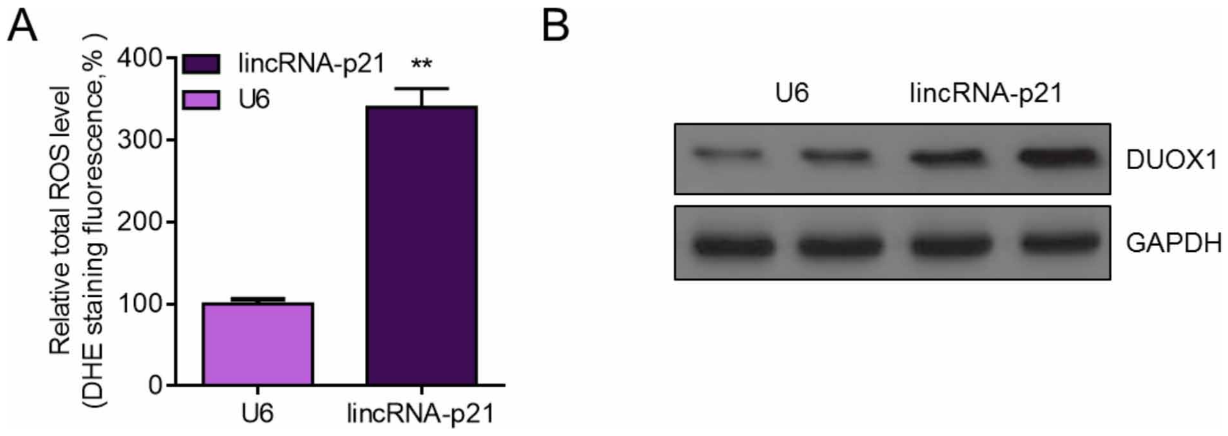

Supplementary Figure S9: LincRNA-p21 regulates oxidative stress. HepG2 cells were infected with indicated lentivirus for 24 hours. **A.** DHE staining was performed to test the total ROS level. Relative total ROS level is shown. **B.** Representative western blot showing lincRNA-p21 increases the level of DUOX1 (dual oxidase 1).

Supplementary Table S1: Primers used for q-PCR

Name	Sense (5′-3′)	Antisense (5′-3′)
lincRNA-p21	CCTGTCCCACTCGCTTTC	GGAACTGGAGACGGAATGTC
IRE1	CAGTGGCCTTTGGTG GGTGTG	GTCTGGGTCTTCGCTGCT CTCCT
CHOP	CACCACTCTTGACCC TGCTTCTCT	GTTTCCTGGTTCTCCCTT GGTCTT
GRP78	TTCGGCACCTCGGTAACCT	GGACTGCGTGATCTCCCTCT
MDM2	TCGTCGGGTGAGGGTACTG	AACCACTTCTTGGAACCAGGT
BAX	CATATAACCCCGTCAACGCAG	GCAGCCGCCACAAACATAC
PUMA	GCCAGATTTGTGAGACAAGAGG	CAGGCACCTAATTGGGCTC
NOXA	CCAAGCCGTGACCAAGGAC	CGCCACATTGTGTAGCACCT
β-actin	GACCTGACTGACTACCTCATGAAGAT	GTCACACTTCATGATGGAGTTGAAGG

Supplementary Table S2: sh RNA sequences targeting human lincRNA-p21.

Sh-RNA	Sequence (5′-3′)
lincRNA-p21–1#	GGAGGACACAGGAGAGGCA
lincRNA-p21–2#	GCCACCCACTCACCACTGG
lincRNA-p21–3#	GCATTGTTGCATCATCATG
lincRNA-p21–4#	CTGCAAGGCCGCATGATGA
